# Aerobic Glycolysis as a Marker of Tumor Aggressiveness: Preliminary Data in High Grade Human Brain Tumors

**DOI:** 10.1155/2015/874904

**Published:** 2015-09-03

**Authors:** Andrei G. Vlassenko, Jonathan McConathy, Lars E. Couture, Yi Su, Parinaz Massoumzadeh, Hayden S. Leeds, Michael R. Chicoine, David D. Tran, Jiayi Huang, Sonika Dahiya, Daniel S. Marcus, Sarah Jost Fouke, Keith M. Rich, Marcus E. Raichle, Tammie L. S. Benzinger

**Affiliations:** ^1^Mallinckrodt Institute of Radiology, Washington University School of Medicine, St. Louis, MO 63110, USA; ^2^Department of Neurological Surgery, Washington University School of Medicine, St. Louis, MO 63110, USA; ^3^Department of Internal Medicine, Washington University School of Medicine, St. Louis, MO 63110, USA; ^4^Department of Radiation Oncology, Washington University School of Medicine, St. Louis, MO 63110, USA; ^5^Department of Pathology & Immunology, Division of Neuropathology, Washington University School of Medicine, St. Louis, MO 63110, USA; ^6^Department of Neurosurgery, Swedish Neuroscience Specialists Ivy Brain Tumor Center, Seattle, WA 98104, USA

## Abstract

*Objectives.* Glucose metabolism outside of oxidative phosphorylation, or aerobic glycolysis (AG), is a hallmark of active cancer cells that is not directly measured with standard ^18^F-fluorodeoxyglucose (FDG) positron emission tomography (PET). In this study, we characterized tumor regions with elevated AG defined based on PET measurements of glucose and oxygen metabolism.* Methods.* Fourteen individuals with high-grade brain tumors underwent structural MR scans and PET measurements of cerebral blood flow (CBF), oxygen (CMRO_2_) and glucose (CMRGlu) metabolism, and AG, using ^15^O-labeled CO, O_2_ and H_2_O, and FDG, and were compared to a normative cohort of 20 age-matched individuals.* Results.* Elevated AG was observed in most high-grade brain tumors and it was associated with decreased CMRO_2_ and CBF, but not with significant changes in CMRGlu. Elevated AG was a dramatic and early sign of tumor growth associated with decreased survival. AG changes associated with tumor growth were differentiated from the effects of nonneoplastic processes such as epileptic seizures.* Conclusions.* Our findings demonstrate that high-grade brain tumors exhibit elevated AG as a marker of tumor growth and aggressiveness. AG may detect areas of active tumor growth that are not evident on conventional FDG PET.

## 1. Introduction

Aerobic glycolysis (AG) refers to glucose utilization in excess of that needed for oxidative phosphorylation, despite sufficient oxygen to metabolize glucose to carbon dioxide and water, and, thus, characterizes different fates of glucose molecules outside of mitochondrial energy production. AG is a marker of a group of metabolic functions which includes biosynthesis of glycogen, proteins, lipids, and nucleic acids; neuroprotection through its role in managing reactive oxygen species and apoptosis, which in the context of the normal brain is involved in synaptic remodeling, learning, and memory; and the generation of energy for membrane pumps [1]. AG has a long history in cancer biology where it also known as the Warburg effect and supports the biosynthetic requirements of proliferating cancer cells [2–6]. Positron emission tomography (PET) using ^18^F-fluorodeoxyglucose (FDG) has been successfully employed in the evaluation of cancer patients under the assumption that an increase in AG will be reflected in an increase in the total glucose consumption (CMRGlu) of the tissue, although false-negative results do occur [7, 8]. It is not known to what degree these false-negative FDG PET scans reflect a discrepancy between CMRGlu and actual AG. Additionally, the measurement of AG may help distinguish tumor from physiological uptake of FDG in normal brain tissue. We sought to investigate this possibility in glial brain tumors by using PET to explicitly measure AG. This is usually accomplished by combining measurements of CMRGlu and oxygen consumption (CMRO_2_) and calculating their molar ratio. If all of the glucose consumed is metabolized to carbon dioxide and water the ratio should be 6 (i.e., 6 moles of oxygen for each mole of glucose). A number less than 6 indicates that fraction of the glucose associated with AG.

In this pilot study, we have measured AG, CMRGlu, CMRO_2_, and cerebral blood flow (CBF) in individuals with high-grade glial tumors and in a control group of age-matched healthy individuals, which was used to identify the regions of the tumors with the highest glycolytic activity. We characterized glucose and oxygen metabolism and cerebral blood flow in regions of the tumors with elevated AG in high-grade glial tumors and evaluated the relationship between AG elevation and tumor growth and clinical course of the disease.

## 2. Materials and Methods

### 2.1. Participants

Fourteen individuals (mean age 50 ± 12 years) with brain tumors and twenty healthy adults (mean age 42 ± 7 years) underwent metabolic PET scans. Healthy individuals were excluded if they had contraindications to MRI, history of mental illness, possible pregnancy, or medication use that could interfere with brain function.

All assessments and imaging procedures were approved by Human Research Protection Office and Radioactive Drug Research Committee at Washington University in St. Louis. Written consent was provided from each participant.

### 2.2. Imaging Acquisition and Analysis

For each participant, a structural MRI scan was performed to provide anatomical information; standard clinical scans were also obtained [9]. MRI scans were obtained in all subjects to guide anatomical localization. High-resolution structural images were acquired using a 3D sagittal T1-weighted magnetization-prepared 180° radio-frequency pulses and rapid gradient-echo (MPRAGE) sequence (TE = 3.93 ms, TR = 1,900 ms, TI = 1,100 ms, flip angle = 20°, 256 × 256 acquisition matrix, 160 slices, 1 × 1 × 1.3-mm voxels). The clinical protocol included T2, FLAIR, and diffusion tensor imaging as previously described [9]. The imaging protocol included 1 mm isotopic 3D T1 weighted imaging before and after intravenous administration of 0.1 mmol/kg of gadolinium contrast (Gadobenate Dimeglumine, Bracco).

^15^O PET scans were performed on a Siemens model 962 ECAT EXACT HR + PET scanner (Siemens/CTI) [10] to measure CBV, CBF, and CMRO_2_ [11–13] and on the same or in one case (participant #6) on the next consecutive day ^18^F-FDG scans were performed on a Siemens Biograph 40 PET/CT or a Siemens Biograph mMR scanner to measure CMRGlu [14] following the same protocols we have described previously [15, 16].

All subjects underwent one FDG scan (to measure CMRGlu [14]) and two sets of three ^15^O scans (CO, H_2_O, and O_2_) to measure CBV, CBF, and CMRO_2_ [11–13]. FDG scans were performed after slow i.v. injection of 5 mCi of FDG. Dynamic acquisition of PET emission data continued for 60 min with 25 5 s frames, 9 20 s frames, 10 1 min frames, and nine 5 min frames. The last 20 min was summed to create the CMRGlu image. Venous samples for plasma glucose determination were obtained just before and at the midpoint of the scan to verify that glucose levels were within normal range throughout the study. CBV was measured with a 5 min emission scan beginning 2 min after brief inhalation of 75 mCi of [^15^O] carbon monoxide in room air as describe previously [11]. Dynamic scans of 3 min with 35 2 s frames, 6 5 s frames, and 8 10 s frames were acquired after injection of 50 mCi [^15^O] water in saline or inhalation of 60 mCi of [^15^O] oxygen in room air, respectively, for CBF and CMRO_2_ measurements [12, 13]. By creating a whole brain time-activity curve, the onset of activity in the brain could be judged exactly, allowing for a consistent selection of the optimal 40 s frame, over which activity was summed. All PET data were reconstructed using a ramp filter (6 mm FWHM) and then blurred to 12-mm FWHM. Subject head movement during scanning was restricted by a thermoplastic mask. All PET images were acquired in the eyes-closed waking state. No specific instructions were given regarding cognitive activity during scanning other than to remain awake.

All images were registered to a common atlas using an affine transformation fitted with in-house software, and all subsequent calculations and comparisons were performed in the atlas space.

#### 2.2.1. Image Analysis

Using an atlas derived brain mask, each individual's CMRGlu, CBV, CBF, and [^15^O] oxygen were scaled to have whole brain means of 1, and then same mode images, if they existed, were averaged. Least-squares regression was used to represent the [^15^O] oxygen scan as a combination of CBF and CBV, and the CBV component was then subtracted from the [^15^O] oxygen scan, yielding an approximation of CMRO_2_, which was itself normalized to 1 [15].

The PET scans were registered to each other and then to the subject's MRI scan, which was in turn registered to an atlas representative target image, corresponding to Talairach space as defined by Lancaster et al. [17]. The PET images were blurred and resampled into atlas space. These registrations and their corresponding transformations were performed with in-house software. A CMRO_2_ parametric image was derived from the ^15^O scans using a previously described method [15, 16], and a CMRGlu image was derived from the FDG scan for each participant [14–16].

#### 2.2.2. AG Measurement

Previously, we have measured and described spatial distribution of AG in the brain of healthy adults and its relationship to accumulation of beta-amyloid in Alzheimer's disease [15, 16]. We evaluated two measures of AG: (1) oxygen/glucose index defined by voxelwise division of relative CMRO_2_ by the relative CMRGlu and (2) glycolytic index, defined by linear regression of CMRGlu on CMRO_2_ and exhibiting the residuals, where positive values represent more AG and negative values represent less glycolysis than predicted by the regression line [15, 16]. These two measures are highly correlated in our data [15, 16]. Oxygen/glucose index images may be noisy in areas of low CMRGlu, as it is in the denominator and the value of the oxygen/glucose index is inversely related to the degree of AG, whereas glycolytic index is positively related to AG. In this study, glycolytic index was obtained to quantify and illustrate AG.

### 2.3. Defining a Tumor Region with Elevated AG

Since the estimation of CMRO_2_, CMRGlu, and AG was achieved by normalizing to whole brain mean [15], which may be problematic for tumor patients due to regional abnormality of metabolism, a separate cohort of healthy control subjects (*n* = 20) was used as the reference. For each individual and image type, a *z*-score image was created using the images of the voxelwise mean and standard deviation of the rest of the control group. No voxels exceeding a score of 4.0 were observed in any image in the control group.

The images in the Patient Group were found to have outlying voxels that significantly affected the results of the normalization and regression procedure used on the control group. To reduce this, an iterative process was used to refine the normalization of each basic PET image. First, a *z*-score image was created by subtracting the control group's corresponding mean image and dividing the result by the control group's standard deviation image. Then, a mask was defined that excluded any voxels above the score of 4.0. The mean values across this mask for both the patient's PET image and the corresponding control group averaged image were obtained, and the patient's image was rescaled to match the two. This was repeated until the results were stable. The final masks were retained for each individual and used to restrict the voxels over which the regressions were performed for creation of CMRO_2_ and AG images. *z*-score images for AG were processed with a threshold of 4.0 and the remaining clusters in the tumor area characterized by increased AG were used to create regions of interest (ROI). Additionally, symmetric contralateral ROIs were generated by flipping these across the transverse axis. These tumor and contralateral ROIs were applied to AG, CMRGlu, CMRO_2_, and CBF images to estimate regional values.

### 2.4. Statistical Analysis

Differences in AG, CMRGlu, CMRO_2_, and CBF in tumor region and symmetric contralateral region were assessed using paired *t*-test. Pearson's correlation coefficient was used to assess the association between CMRGlu, CMRO_2_, and CBF in tumor region with increased AG and in symmetric region in contralateral hemisphere, both unadjusted and adjusting for age and the volume of the region. Survival rates were calculated by the Kaplan-Meier method and the differences between the curves were evaluated by Log Rank and Tarone-Ware tests. *p* values < 0.05 were used to indicate statistical significance.

## 3. Results

Demographic characteristics of the participants and the history of surgical and medical treatment are presented in Table 1. We found that newly diagnosed and recurrent high-grade glial brain tumors demonstrate significantly elevated AG.  Table 2 shows individual values for different PET measures estimated in the area with elevated AG in the tumor region and in the same area of the contralateral hemisphere. There are no data for participants ##10–13 in Table 2 because these tumors did not demonstrate significant increase in AG and no tumor AG-based ROIs were defined. In most cases, AG PET studies were done during follow-up after surgery, radiation, and chemotherapy, status of the tumor (progression or stable course) was defined based on visual interpretation of serial MR scans, and no recent histological information was available to correlate it to AG and other PET measures in corresponding areas.

In general, elevated AG associated with substantial oxygen hypometabolism was the typical pattern for all tumors with progressive growth (Table 2). AG both visually and quantitatively (Table 2) demonstrated the metabolically active part of the tumor much better than CMRGlu, which may be unchanged in many cases, or CMRO_2_, which does not efficiently distinguish the solid part of the tumor from the adjacent areas with edema. In individuals with elevated AG in the tumor region (Table 2), AG was substantially higher (*p* < 0.0001), CMRGlu was not different (*p* = 0.084), and CMRO_2_ (*p* < 0.0001) and CBF (*p* = 0.016) were lower in the tumor compared to the symmetric region in contralateral hemisphere. Physiological coupling between CMRGlu, CMRO_2_, and CBF was disrupted in the tumor region but preserved in the intact region in contralateral hemisphere. Specifically, strong positive correlation was demonstrated between CMRGlu and CMRO_2_ (*r* = 0.907, *p* = 0.001), CMRGlu and CBF (*r* = 0.845, *p* = 0.004), and CMRO_2_ and CBF (*r* = 0.938, *p* < 0.001) for the symmetric region in contralateral hemisphere; however, no correlation was found for the tumor region (*r* = 0.394, *p* = 0.294 for CMRGlu and CMRO_2_; *r* = 0.393, *p* = 0.296 for CMRGlu and CBF; and *r* = 0.509, *p* = 0.161 for CMRO_2_ and CBF). The same associations were demonstrated after regressing age and size of the ROI.

**Figure 1:**
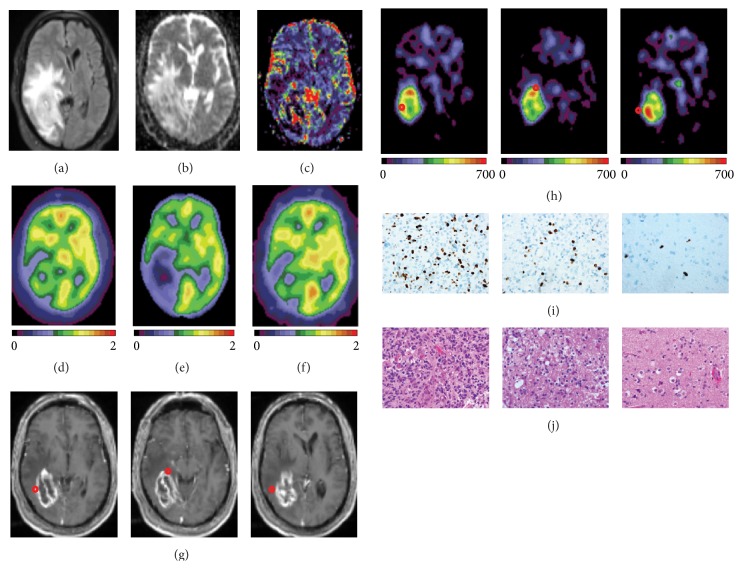
Coregistered PET and MR images from an individual with glioblastoma (participant #1). ((a)–(f)) MR and PET images of the same tomographic slice demonstrating the tumor region: (a) FLAIR MR image; (b) Apparent Diffusion Coefficient (ADC) diffusion-weighted MR image; (c) Cerebral Blood Volume (CBV) perfusion-weighted MR image; (d) CMRGlu PET image; (e) CMRO_2_ PET image; (f) CBF PET image; ((g)-(h)) biopsy sites (showed with red circles) in tumor (left and middle) and peritumoral (right) regions: (g) T1-weighted MR images with contrast (Gadolinium) enhancement; (h) AG PET images. ((i)-(j)) Histopathology for the corresponding biopsy sites: (i) Ki-67 labeling and (j) Hematoxylin and eosin stained sections.

In one case of newly diagnosed glioblastoma (World Health Organization, WHO, grade IV; participant #1, Figure 1), PET imaging was performed shortly prior to surgery, and stereotactic coordinates from biopsied specimens from several regions were available. When we measured AG in the standard spheres (5 mm radius) created around the center of coordinates for biopsies we found that the specimen taken from the areas of increased AG (Figure 1(h)) demonstrated a presence of a glial neoplasm with high proliferative activity (frequent mitotic figures, high percent of neoplastic tissue, and high Ki-67). Moreover, the tumor region with higher Ki-67 showed higher AG (Ki-67 = 25.8, AG = 390; Figures 1(g) and 1(h), left) than tumor region with lower Ki-67 (Ki-67 = 6.9; AG = 307; Figures 1(g) and 1(h), middle). Some atypical appearing cells were observed in specimen taken from brain areas outside of significant increase in AG (Ki-67 = 0.2; AG = 88; Figures 1(g) and 1(h), right). Hematoxylin and eosin stained sections show changing cellular density (Figure 1(j)) along with the corresponding Ki-67 labeling indices (Figure 1(i)). While the infiltrative edge of malignant glioma (Figure 1(j), right) is comprised of only scattered neoplastic cells, the actual tumor mass is represented in images in Figure 1(j) (left and middle) albeit with variable areas of cellular density (middle is less dense as compared to left). The area with elevated AG demonstrated substantial decrease in CMRO_2_ (Figure 1(e)); however, CMRGlu (Figure 1(d)) and CBF (Figure 1(f)) were not changed compared to the contralateral side (Figure 1, Table 2).

**Figure 2:**
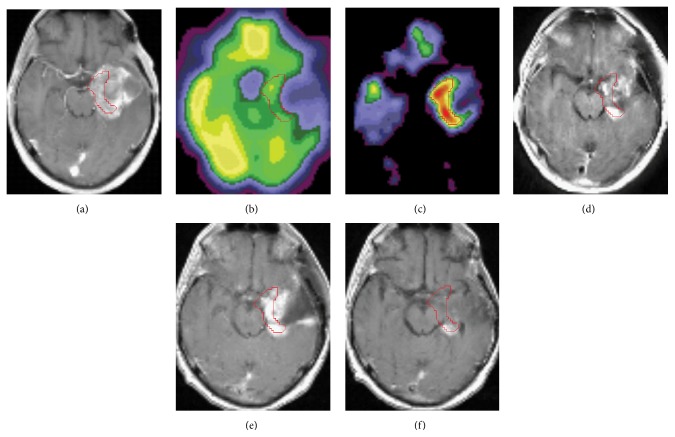
Coregistered PET and MR images from an individual with PNET WHO stage IV (participant #6). Red line delineates the area with the largest increase in AG. ((a)–(c)) MR and PET images at the time of the PET study and 1 day before surgery: (a) T1-weighted image with contrast (Gadolinium) enhancement; (b) CMRGlu PET image; (c) AG PET image. ((d)–(f)) T1-weighted MR images with contrast (Gadolinium) enhancement after partial surgical resection: (d) five days and (e) six weeks after surgery; (f) five months after surgery followed by radiation and chemotherapy.

**Figure 3:**
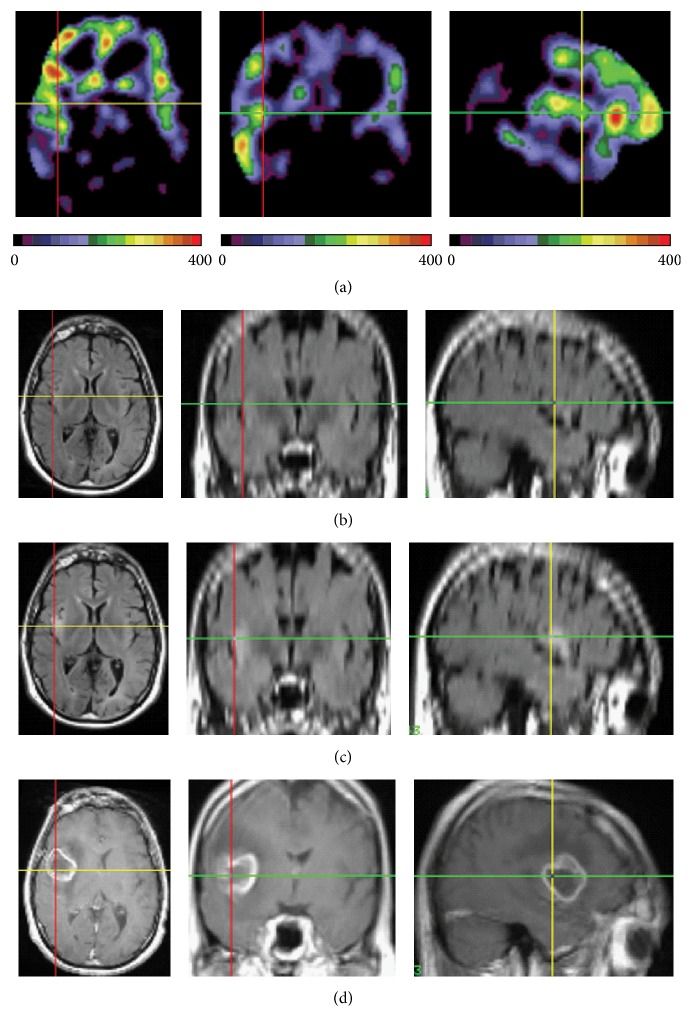
PET and MRI axial (left row), coronal (middle row), and sagittal (right row) images from an individual with familial Alzheimer's disease and GBM (participant #13). (a) AG PET image showing regional asymmetry with higher AG in the right hemisphere. (b) FLAIR MR image at the time of PET study, showing no evidence of abnormality. (c) FLAIR MR image a year later demonstrating first evidence of abnormal signal; (d) MR T1-weighted image with (Gadolinium) contrast enhancement 21 months after PET study, showing GBM.

Our findings indicate that AG image correlate well with the clinical course of the disease (Figures 2 and 3).  Figure 2 illustrates the findings in primitive neuroectodermal tumor (PNET) WHO grade IV, postgross resection, radiotherapy, and chemotherapy (participant #6), which demonstrated MR and PET evidence of the disease progression, although only a part of a large contrast enhancing region was highly glycolytic (Figures 2(a) and 2(e)). Shortly after the PET study, tumor was partially resected, although the most glycolytic part could not be safely removed due to its location (Figure 2(d)) and the tumor subsequently progressed 6 weeks after the surgery (Figure 2(e)). After additional chemotherapy temporal stabilization of clinical course and reduction of contrast enhancement (Figure 2(f)) were observed; however, several months later tumor progressed substantially with fatal clinical deterioration.

Figure 3 shows the PET and MRI images of 43-year-old individual with familial (early-onset) Alzheimer's disease (participant #13, Table 1). At the time of the PET study this participant had mild-to-moderate dementia (clinical dementia rating or CDR score 0.5) [18], there were no prominent structural changes on his MRI scans (Figure 3(b)), and the PET scans demonstrated hypometabolism in precuneus and posterior cingulate brain cortex, which is consistent with AD. Remarkably, there was an unexpected asymmetry in AG, which was higher in the right frontal cortex (Figure 3(a)). The asymmetry in AG and also in CMRO_2_ was also noticed in several cortical regions and caudate with regional FreeSurfer [19, 20] analysis (Table 3). A year later, his follow-up MR fluid attenuation inversion recovery (FLAIR) scan suggested interval development of abnormal hyper intense signal regions in right insular and frontal cortex (Figure 3(c)), and during following 9 months the patient progressively deteriorated clinically along with the development of multiple MR contrast enhancing lesions in the right hemisphere (Figure 3(d)), which at autopsy were verified as glioblastoma.

**Figure 4:**
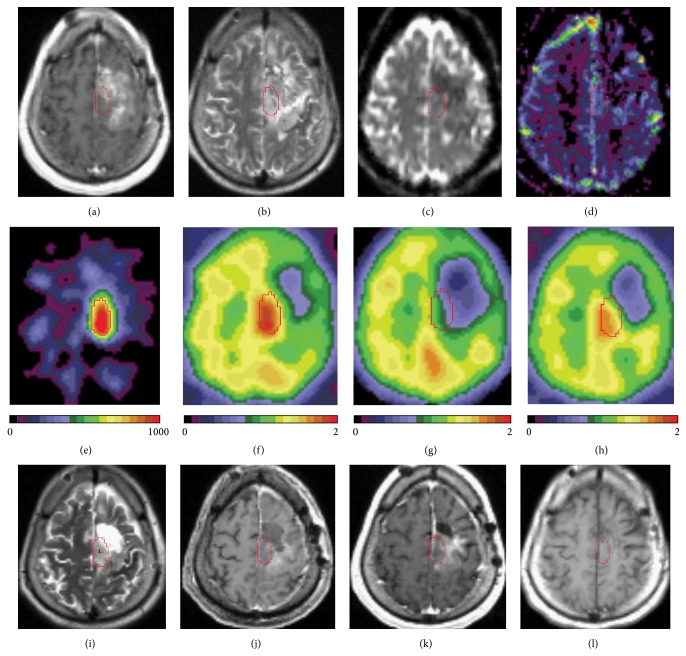
Coregistered PET and MR images from individual with anaplastic oligodendroglioma WHO grade III (participant #14). ((a)–(d)) MR images obtained at the time of PET study. (a) T1-weighted MR image with contrast (Gadolinium) enhancement; (b) T2-weighted MR image; (c) Apparent Diffusion Coefficient (ADC) diffusion-weighted MR image; (d) Cerebral Blood Volume (CBV) perfusion-weighted MR image; (e) AG PET image; (f) CMRGlu PET image; (g) CMRO_2_ PET image. (h) CBF PET image; (i) T2-weighted MR image 1 day after surgery (2 days after the PET study); ((j)–(l)) Follow-up T1-weighted images with contrast (Gadolinium) enhancement obtained 2 days (j), two months (k), and 14 months (l) after the PET study.

In one case of long standing recurrent anaplastic oligodendroglioma WHO grade III (participant #14, Figure 4, Table 2), AG was substantially increased in a large part of the left frontal (mainly motor) cortex (Figure 4(e)) partially overlapping with contrast enhancing area (Figure 4(a)). This AG increase was associated with elevated CMRGlu (Figure 4(f)) and CBF (Figure 4(h)) and very moderate decrease in CMRO_2_ (Figure 4(g)), so that all PET measures were numerically the highest compared to that in all other participants (AG = 464; CMRGlu = 1.40; CMRO_2_ = 0.88; CBF = 1.13). Importantly, this individual experienced recurrent focal seizures in right hand and leg resistant to antiepileptic treatment. They did not disturb the individual unduly; however, they were very frequent, and a neurosurgeon noticed three or four episodes during the space of the office visit a month before his PET study. Shortly after the PET study, this participant underwent partial resection of the tumor leaving the most part of motor cortex with elevated AG (Figures 4(i) and 4(j)), and some improvement of his seizure frequency was reported two months later from 12–14 times per day to 5-6 times per day. Then, the participant underwent IMRT and chemotherapy and his seizures ceased and stable course of the disease has been observed with no MR evidence of tumor progression for 2 years of follow-up.

**Figure 5:**
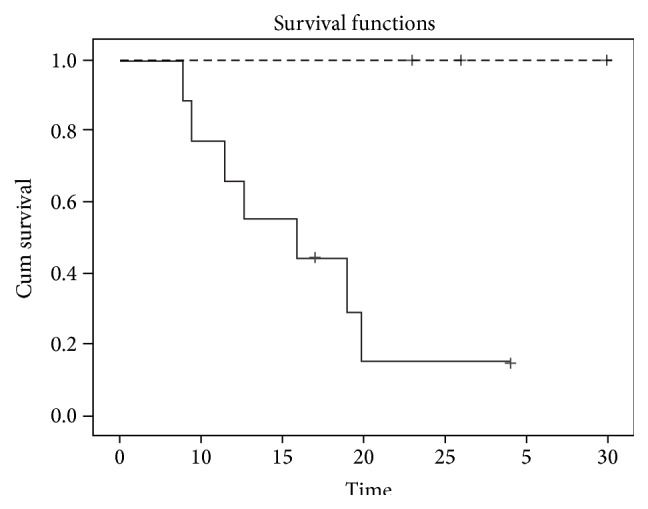
Longitudinal survival (time in months) in individuals with tumors associated with elevated AG (solid) and those without significant increase in AG (dashed).

During the follow-up period, participants ##1–6 and 9 (Table 1) demonstrated progression of the disease and expired within 4–15 months after PET studies. Participant #7 underwent two surgeries followed by radiation and chemotherapy and was stable at the time the paper was submitted. Participant #8 demonstrated stabilization of the disease after chemotherapy. No progression was noted in participants ##10–12 (Table 1) during the follow-up of 18 to 30 months. The Kaplan-Meier survival curve analysis demonstrated that individuals with elevated AG have lower survival rate compared to individuals without elevated AG (*p* = 0.034 by Log Rank test and *p* = 0.042 by Tarone-Ware test) (Figure 5).

## 4. Discussion

Our findings indicate that metabolic PET studies with explicit measurements of AG provide information which cannot be obtained with conventional neuroimaging techniques. Specifically, we demonstrated that high-grade brain tumors exhibit elevated AG associated with decreased CMRO_2_, but usually no significant changes in CMRGlu were noted. Despite decreases in CMRO_2_ being a prominent feature of the Warburg effect, this measure alone was not very helpful for delineation of the most active part of the tumor, because CMRO_2_ image does not allow distinguishing hypometabolism in the proliferating tumor from that in the adjacent areas with necrosis or edema. In an individual with newly diagnosed glioblastoma who underwent histological sampling of the tumor, we observed elevated AG changing in parallel with the rate (index) of proliferation. In tumors demonstrating no evidence of recurrence or progression, AG was not significantly elevated and there were no changes in CMRGlu or CMRO_2_. In these cases, the clinical course during the follow-up was more favorable than in tumors demonstrating elevated AG. Additional studies will be needed to assess the diagnostic accuracy of AG measures to distinguish viable tumor from nonneoplastic processes such as radiation necrosis.

A unique finding in our study is the observation of AG abnormalities in an individual with the familial AD who later was diagnosed with glioblastoma. These abnormalities manifested as an asymmetry of AG in the absence of any pathological signs on MR. Only a year later FLAIR MR scan showed some initial changes; however, afterwards clinical deterioration and progression of changes on MR were rapid. This observation raises the possibility that metabolic alterations including elevated AG may occur early in brain tumor formation and warrants further investigation.

The upregulation of AG (Warburg effect) provides a selective advantage for the survival and proliferation of tumor cells and many mechanisms serve these purposes. One mechanism by which cancer cells establish the Warburg effect is via transcriptional upregulation of glycolytic enzymes. Elevated expressions of glucose transporters and glycolytic enzymes are found in many cancers and may contribute to tumor growth [21–23]. The hypoxia-inducible factor 1 (HIF1) has recently been shown to actively suppress mitochondrial respiration by directly upregulating the expression of the gene encoding pyruvate dehydrogenase kinase 1 (PDK1) [24, 25]. Initially HIF-1 was believed to be a transcription factor involved in hypoxia; however, it has more recently been shown to be active in normoxic conditions [26, 27]. Of note, the highest increases in AG in glial tumors observed in our study (e.g., see Figure 1) were located in the periphery of the main tumor mass and not in the core of the tumor, where hypoxia could be more likely expected.

M2 isoform of pyruvate kinase is expressed in both cancer cells and normal proliferating cells [28] and it promotes both aerobic glycolysis and anabolic metabolism [29]. Glycolytic enzyme hexokinase 2 is aberrantly expressed in GBMs being an important mediator of aerobic glycolysis and providing a proliferative and cell survival advantage [30]. The tumor suppressor p53, involved in the DNA damage response and apoptosis, is another important transcription factor that regulates glycolysis, oxidative phosphorylation, and pentose phosphate pathway activity [31]. The loss of p53 expression in tumor cells may facilitate the Warburg effect by simultaneously increasing AG and decreasing oxidative phosphorylation. Thus, all these mechanisms upregulate glycolysis, and lower levels of mitochondrial activity lead to a decrease in both reactive oxygen species production and the propensity of mitochondria to depolarize the events that trigger apoptosis. PET studies of AG combined with guided biopsy with detailed pathological evaluation and characterization of biological, chemical, and genetic profiles of biopsies will be necessary to demonstrate the mechanisms involved in metabolic management of human brain tumor development.

We believe that changes in AG and other PET measures associated with seizure activity in one of our participants (#14) were originated from a mixture of tumor and nontumor cells but primarily from nontumor cells. Increase in AG in this oligodendroglioma grade III was mostly beyond the contrast enhancing area and more substantial than in more aggressive tumors in other participants, and although most of this area remained after surgery, there were no MR signs of progression in the follow-up period, while clinical improvement after radiation and chemotherapy was obvious, and seizures first decreased in frequency and then ceased completely. Metabolic pattern of the lesion was another unique feature. Increase in AG was associated with substantial elevation of CMRGlu in parallel with CBF, but with no marked changes in CMRO_2_. Actually, the pattern was very similar to what we see in response to physiological activation (e.g., visual stimulation) [14], although seizures have much more powerful impact. Thus, here we demonstrated that AG may be elevated not only in growing tumor but also due to extreme activation of neighboring tissue by intractable tumor-associated epileptic activity. Evaluation of a pattern of changes in AG, glucose, and oxygen metabolism allows distinguishing of potential sources of elevated AG. Of note, data on AG in seizures are very limited due to the difficulty of performing PET studies during the ictal phase, and we believe that here we are presenting the first measurement of AG evaluated during the continuing focal epileptic activity.

There are several limitations to our study. We have a small and highly heterogeneous group of tumors arising from different cells and at different stages. We have insufficient number of observations to distinguish whether elevated AG is different between grades III and IV tumors, although our data suggest that it is not the grade itself but proliferative activity which defines the level of AG. Our PET studies were cross sectional done either before or after surgery and radio- or chemotherapy. In most cases, pathological characteristics of the tumor were not provided at the time of our PET studies; therefore, we were not able to evaluate the relationship of AG to Ki-67 or other measures of proliferative activity in all participants. Our PET measurements of AG and CMRO_2_ are technically demanding and require ^15^O inhalation and injection which are not easily available at PET centers, whereas reliable alternative MR techniques are still under development.

## 5. Conclusions

Our pilot study on a small cohort provided several unique observations suggesting a critical role of AG in human brain tumors. Our findings demonstrated that primary brain tumors exhibit AG as a marker of tumor growth and aggressiveness and indicated that AG can be used to detect areas of active tumor growth which may otherwise be missed by conventional FDG PET suggesting that explicit measurements of AG might increase the efficacy of PET imaging in the management of oncologic patients. Longitudinal PET studies of brain metabolism and AG are needed on a large cohort of individuals with various brain tumors and metastases to verify its role in the tumor growth and evaluate its efficacy in tumor grading and differential diagnosis, as well as its potential for prognosis, treatment planning, and evaluation of treatment response and tumor recurrence.

## Figures and Tables

**Table 1:** Demographic characteristics and medical history.

Participant	Age	Gender	Pathology, WHO grade	Location	Surgery prior to the study	Treatment prior to the study	Status at the time of study	Aerobic glycolysis
1	57	M	GBM, IV	R temporooccipital	None	None	Newly diagnosed	Elevated
2	71	M	GBM, IV	R temporal	Resection	IMRT, TEM	Recurrent	Elevated
3	69	M	GBM, IV	L temporoparietal	Resection	IMRT, TEM	Recurrent	Elevated
4	49	M	GBM, IV	L temporal	Resection	IMRT, TEM, and Avastin	Recurrent	Elevated
5	58	M	GBM, IV	R occipital	Resection	IMRT, TEM	Recurrent	Elevated
6	42	M	PNET, IV	L temporal	Resection	IMRT, Vincristine, and Cisplatin	Recurrent	Elevated
7	43	M	AA, III	R frontal	Resection	IMRT, TEM	Recurrent	Elevated
8	57	F	AOA, III	L temporooccipital	Biopsy	IMRT, TEM	Progression	Elevated
9	56	M	AOA, III	L parietal	Biopsy	IMRT, TEM	Stable disease	Elevated
10	57	M	PNET, IV	R temporal	Resection	WBRT, TEM	Residual/recurrent	Not elevated
11	29	M	AO, III	L frontal	Resection	IMRT, TEM	Stable disease	Not elevated
12	30	F	AA, III	L frontoparietal	Biopsy	None	Newly diagnosed	Not elevated
13	43	M	FAD; GBM, IV	R frontal	None	None	FAD	Asymmetry
14	40	M	AO, III	L frontal	Resection	IMRT, TEM	Recurrent	Elevated

M, male; F, female; WHO, World Health Organization; GBM, glioblastoma; PNET, primitive neuroectodermal tumor; AA, anaplastic astrocytoma; AOA, anaplastic oligoastrocytoma; AO, anaplastic oligodendroglioma; FAD, familial Alzheimer's disease; R, right; L, left; IMRT, intensity-modulated radiation therapy; WBRT, whole brain radiation therapy; TEM, Temozolomide.

**Table 2:** PET metabolic and blood flow measures in AG-defined ROIs in the tumor and in the symmetric region of contralateral hemisphere.

Participant	AG		CMRGlu		CMRO_2_		CBF	
	Tumor	Contralateral	Tumor	Contralateral	Tumor	Contralateral	Tumor	Contralateral
1	358	22	1.01	1.06	0.59	1.10	0.87	0.99
2	356	−37	0.93	1.06	0.49	1.09	0.83	1.07
3	374	38	1.03	1.18	0.60	1.21	0.80	1.14
4	350	−6	0.96	1.02	0.57	1.06	0.91	0.99
5	311	95	0.89	0.81	0.54	0.69	0.80	0.75
6	289	16	0.97	1.10	0.57	1.02	0.90	1.08
7	413	69	1.12	1.03	0.47	0.87	0.79	0.80
8	289	−5	0.81	0.88	0.50	0.92	0.64	0.93
9	183	59	0.69	0.79	0.47	0.71	0.79	0.77

**Table 3:** PET metabolic measures in FreeSurfer ROIs corresponding to pathologically verified tumor in the right frontal and insular region and in contralateral (left) hemisphere in individual with familial AD and GBM (participant #13).

FreeSurfer region	AG		CMRGlu		CMRO_2_		CBF	
	Right	Left	Right	Left	Right	Left	Right	Left
Rostral middle frontal	171	118	1.11	1.10	0.95	1.00	0.96	0.96
Transverse temporal	206	113	1.36	1.33	1.21	1.30	1.19	1.26
Pars orbitalis	167	19	1.04	0.99	0.87	0.99	0.88	0.89
Pars triangularis	200	71	1.16	1.15	0.98	1.12	1.00	1.00
Pars opercularis	162	155	1.28	1.26	1.17	1.15	1.21	1.12
Insula	60	37	1.19	1.18	1.19	1.20	1.25	1.20
Caudate	158	76	1.08	1.09	0.94	1.05	0.96	1.00
Precuneus	27	−24	1.05	1.09	1.10	1.12	1.08	1.04
